# Overweight in Rural *Quilombola* and *Non-quilombola* Adolescents From the Northeast of Brazil

**DOI:** 10.3389/fnut.2020.593929

**Published:** 2021-02-09

**Authors:** Stefanie M. C. Cairo, Camila S. S. Teixeira, Tainan O. da Silva, Etna K. P. da Silva, Poliana C. Martins, Vanessa M. Bezerra, Danielle S. de Medeiros

**Affiliations:** ^1^Program of Post-Graduation in Collective Health, Multidisciplinary Institute of Health, Federal University of Bahia, Vitória da Conquista, Brazil; ^2^Program of Post-Graduation in Public Health, Institute of Collective Health, Federal University of Bahia, Salvador, Brazil; ^3^Program of Post-Graduation in Public Health, Faculty of Medicine, Federal University of Minas Gerais, Belo Horizonte, Brazil

**Keywords:** adolescents, African continental ancestry group, Brazil, overweight, rural communities, vulnerable population

## Abstract

**Introduction:** Overweight is an emerging problem among children and adolescents that leads to the development of several morbidities and health risks. Overweight occurs differently in different populations, especially in vulnerable groups like the rural and *quilombola* communities (an African-descendant population). This study aimed to estimate the prevalence of overweight and to investigate the possible associated factors in rural adolescents living in both *quilombola* and *non-quilombola* communities in Northeast Brazil.

**Methods:** This study is a population-based cross-sectional study with a household approach carried out in 2015 with 390 adolescents (age 10–19 years) living in rural *quilombola* and *non-quilombola* communities. The nutritional status was gauged using z-scores calculated for body mass index (BMI) and varies with gender and age. Prevalence ratios (PRs) and 95% confidence intervals (95% CIs) were used to establish associations between the results and explained variables. The multivariate analysis followed a model with a hierarchical entry of covariables controlled by gender and age.

**Results:** The study showed that 18.5% of rural adolescents were overweight, of which 17.9% were *quilombolas* and 19.0% were *non-quilombolas*. A significant difference in overweight between the samples was not found. In the multivariate-adjusted model, age ≥16 years (PR: 0.51; 95% CI: 0.28–0.95), the habit of having regular breakfast (PR: 0.58; 95% CI: 0.35–0.98), and process of attending school (PR: 0.35; 95% CI: 0.17–0.71) were associated with a lower prevalence of overweight. Stationary screen time, in contrast, was associated with a higher prevalence (PR: 1.61; 95% CI: 1.05–2.46). The process of attending school was associated with a lower prevalence of overweight (PR: 0.26; 95% CI: 0.09–0.69), even for the *quilombolas*.

**Conclusions:** A low prevalence of overweight was identified in rural adolescents. Overweight was significantly associated with the habit of having regular breakfast, older age, stationary screen time, and the process of attending school. The results reveal that school is a potential space for health promotion interventions, specifically in the most vulnerable rural regions, such as the *quilombola* communities. Besides, the study emphasizes the importance of adopting a healthy lifestyle early in life, including cultivating the habit of having regular breakfast and reducing stationary screen time.

## Introduction

Adolescence is a period in which physical and psychological changes that contribute to vulnerability occur in one's life cycle (1). During this period, there is a great change in body composition due to many factors, such as eating habits, physical activities, age, and gender (2).

In the adolescent population, an increase in sedentariness and a decrease in physical activity are frequently observed; poor eating habits, including a high intake of ultra-processed foods, long intervals between meals, low intake of fruits and vegetables, and replacement of traditional meals with fast food are common (3, 4). These behaviors contribute to weight gain and metabolic alterations, besides being risk factors for nutritional deficiencies and non-communicable chronic diseases. Poor eating habits and decreased physical activity have both short- and long-term effects on adulthood (5).

Overweight and obesity are considered the most direct consequences of these bodily changes and emerging problems among children and adolescents (6). A study that gathered and analyzed data from 2,416 population-based studies included 31.5 million children and adolescents worldwide between 5 and 19 years of age and showed that the prevalence of obesity increased from 0.7 to 5.6% in girls and 0.9 to 7.8% in boys between 1975 and 2016 (7). Obesity was prevalent in more than 30% of children and adolescents living in Oceania and around 20% of children and adolescents in Polynesia and Micronesia, the Middle East, north of Africa, the Caribbean, and the USA (7). In Brazil, results of the *Pesquisa Nacional de Saude do Escolar (PeNSE)*, a national school-based health survey carried out in 2015, showed that 23.7 and 7.8% of adolescents between 13 and 17 years of age were overweight or obese, respectively, in the capitals and metropolitan areas of the country (8). In the Northeast region, 20.5% were overweight and 6.4% were obese (8).

However, such problems can occur in different ways in different populations. Considering the social and economic contexts, especially in the urban/rural Brazilian scenario, access to education and health services in the rural area affects people in all age groups (9). In the traditionally vulnerable groups like the *quilombola* communities, the health conditions are poor (10), especially concerning bad eating habits and overweight (11). *Quilombola* communities are distributed throughout Brazil, and most of them are located in rural areas of the Northeast (12). These communities still live with social inequities (13, 14) and suffer the effects of historical racial segregation and expropriation (12).

Among the *quilombola* adolescents, discrimination can influence growth issues, including health, in a negative way (15). A study carried out in the Northeast of Brazil showed differences in the intake of healthy food between *quilombola* and *non-quilombola* rural adolescents. *Quilombola* adolescents had a lower intake of milk, vegetables, and fruits when compared to the *non-quilombola* adolescents (16), which may adversely impact the weight of this population.

Considering that adolescence is a vulnerable period for human development, healthy habits, and learned behaviors during this period can have long-term consequences extending to adulthood. Therefore, it is necessary to investigate the occurrence of and factors causing overweight in this age group. Identifying overweight in rural adolescents can help prevent health problems, specifically among the *quilombolas*. Therefore, this study aimed to estimate the prevalence of overweight and the factors associated with it in rural adolescents from the Northeast of Brazil.

## Methods

### Study Design, Population, and Sample

This is a population-based cross-sectional study with a household approach carried out with adolescents, 10–19 years old (17), from rural communities of Vitoria da Conquista, State of Bahia, Northeast of Brazil. The study analyzes data from the research “*Adolescer: saude do adolescente da zona rural e seus condicionantes*” (Adolescer: Rural Adolescent Health and Its Conditioning) carried out in 2015.

To carry out the population estimate, we collected data from Brazilian Primary Health Care forms used by the community health workers during the household visits. The Program of Community Health Workers covered 97.4% of the rural area of Vitoria da Conquista at the time of the study.

We used a sampling strategy that took into account the territorial extension of rural communities and populations of adolescents to ensure viability and representativeness of the research. The sampling principles used were as follows: (1) the number of households was selected proportionally to the number of adolescents per community and (2) only one adolescent was interviewed per household. Moreover, in order to obtain valid estimates for *quilombola* and *non-quilombola* populations, the sample size was calculated separately for each stratum.

We calculated the sample size using the following criteria: a prevalence of 50%, given the heterogeneity of the events measured in the main project; an accuracy of 5%; a confidence level of 95%; a design effect equal to 1.0; and an addition of 15% for possible losses. However, as only one adolescent per household was interviewed and because the number of households was smaller for the *quilombola* communities, 7.1% of losses were added to the *quilombola* stratum. OpenEpi, version 3.01 (open source epidemiological statistics for public health) (18), was used for this estimation. The presence of severe mental disorders among adolescents was used as an exclusion criterion.

Sampling for *non-quilombola* adolescents was carried out in two stages: (1) random selection of households with adolescents according to the proportional distribution of adolescents per community and (2) random selection of adolescents in each household. For the *quilombola* sample, only random selection of adolescents in each household was used. All adolescents had the same probability of inclusion in the study.

The research was approved by the Institutional Review Board of the Federal University of Bahia *(Comite de Etica em Pesquisa com Seres Humanos da Universidade Federal da Bahia*–*Instituto Multidisciplinar em Saude*–*Campus Anisio Teixeira)* under rule number 639.966. The participants received information about the research objectives and data confidentiality prior to study initiation. They were required to read and sign the free informed consent form and informed consent form for adolescents under 18 years of age.

### Data Collection Survey

For data collection, a semistructured survey was formulated based on questionnaires from national inquiries, such as *Pesquisa Nacional de Saude do Escolar (PeNSE)* by the national school-based health survey and *Pesquisa Nacional de Saude (PNS)* by the National Health Survey (19, 20). The software, Questionnaire Development System (QDS^TM^; NOVA Research Company) version 2.6.1, was used for constructing and visualizing the questionnaires.

The survey was divided into two parts: (i) the first part was answered by the adolescents or their legal representatives and addressed general characteristics regarding the residence, income, and schooling of the householder; (ii) the second part was answered only by the adolescents (in the absence of their parents and in a comfortable place that guaranteed confidentiality of the answers and minimized potential information bias) and addressed the characteristics of the adolescents, support from society, characteristics of their work, lifestyle, perception of health conditions and self-image, deficiencies (intellectual, physical, hearing, and visual), use of illicit drugs, accidents and violence, sexual and reproductive health, oral health and hygiene, and use of health services.

The final version of the survey was evaluated after the pretests and a pilot study. The vocabulary and response options were adapted and normalized for the rural context while retaining the original validated structure for better understanding and ensuring comparability of information. Pretests were performed, and the survey was normalized based on (1) language, (2) sequence of and coherence between questions, (3) instructions on questions to be skipped, and (4) the time required to finish the survey. After the pilot study, new changes were made to the language to obtain the final version.

The pilot study was carried out in December 2014 in a rural community that was originally not a participant in the main study; the population was equal to 10% of the sample size of the main study. The exclusion criteria included situations in which both the adolescents or their legal representatives were unable to answer the questions because they were drunk at the time of data collection or had serious mental health problems and cognitive interference.

### Data Collection

The data were collected between January and May 2015. To ensure the credibility of the data, interviews were repeated for 5% of the samples within 7 days after the interview. The interviewers received training to conduct the interviews and used portable computers (HP *Pocket* Rx5710).

The interviewing team was composed of 15 undergraduate students from nursing, pharmacy, nutrition, medicine, and psychology branches, who had previously participated in rural research projects. They received a 20-h training conducted by the coordination team and focused on the following aspects: approaching adolescents, conducting interviews, ethical aspects, measuring anthropometric measures, handling and using equipment and software, mapping territories, and identifying households.

The anthropometric measurements were done according to *Normas Técnicas do Sistema de Vigilância Alimentar e Nutricional (SISVAN) do Brasil—*Brazilian Technical Rules of Diet and Nutrition Surveillance System (21). The weight was measured in kilograms (kg) for barefoot individuals wearing light clothes using a portable scale (*Marte*, model LC 200pp) having a maximum capacity of 200 kg and precision of 0.05 kg. The height was measured in centimeters (cm) for barefoot individuals in a standing posture using a portable stadiometer (*CauMaq*, model est-22) with lateral readings, a maximum height of 2 m, and graduations in millimeters (mm).

### Variables

The nutritional status was gauged by calculating the body mass index (BMI) and height deficit. BMI was classified according to the curves proposed by World Health Organization (WHO) using *WHO AntroPlus* version 1.0.4. The software calculates Z-scores for BMIs based on gender and age (22). The cutoff points include low weight (−2 > z-score ≥ −3), eutrophy (+1 ≥ z-score ≥ −2), overweight (+2 ≥ z-score > +1), and obesity (z-score > +2) (23). Based on the cutoff points, overweight and obesity were identified through responses in the questionnaire; the responses were categorized as “*yes*” (z-score > +1) and “*no*” (z-score ≤+1). The height deficit for age was evaluated with the following cutoff points: deficit present (−2 > z-score ≥ −3) and deficit absent (z-score ≥ −2) (23).

The independent variables were gender; age; race/color (non-black—white, Asian, Brazilian indigenous; black—mulatto and black); school years; economic level (A/B and C/D— *Associacao Brasileira de Pesquisas e Mercados*—Brazilian Association of Market Research) (24); currently attending school; family composition; the number of close friends; experience with bullying; the practice of physical activities (active, ≥300 min/week; inactive, <300 min/week) (25); stationary screen time (the time spent in front of the TV daily >2 h) (26); regular intake of unhealthy food (intake of typically unhealthy food ≥5 days per week, such as processed meats, crackers, cookies, fried chips, dainties, and soda); breakfast intake (frequency ≥5 days per week) (27); habit of eating while watching TV (frequency ≥5 days per week) (27).

### Statistical Analysis

Simple frequencies were calculated, and the differences between *quilombola* and *non-quilombola* samples were compared using Pearson's chi-square or Fisher's exact test. Differences between proportions were assessed using Pearson's chi-square or Fisher's exact test. The prevalence ratio (PR) and 95% confidence interval (95% CI) were used to estimate the association between the results and explained variables. Poisson regression with robust variance was used for the multivariate model to obtain better estimates of PR for very frequent results.

**Figure 1:**
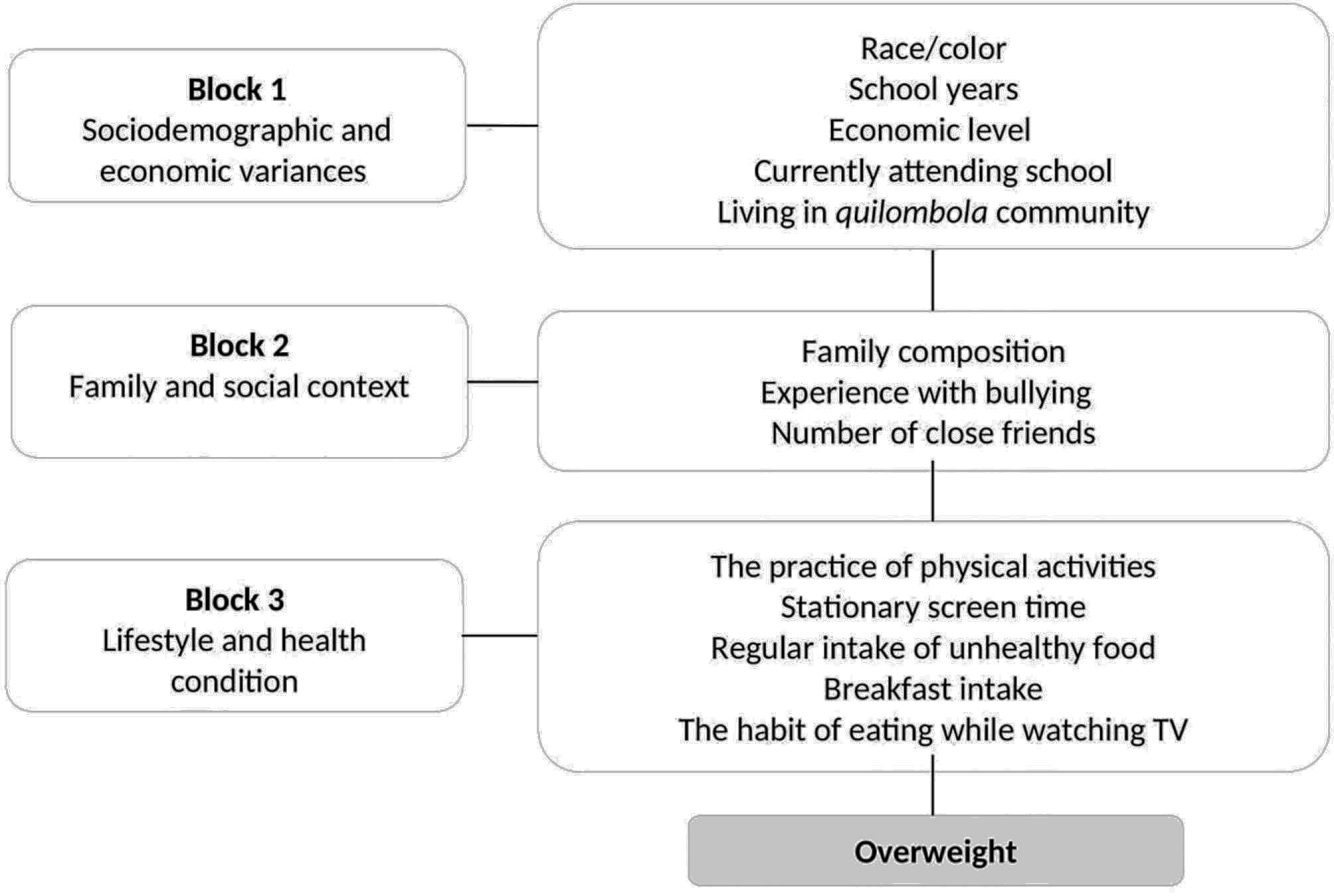
Conceptual model of multivariate analysis for overweight among *quilombola* and *non-quilombola* rural adolescents.

The multivariate analysis followed the model of hierarchical entry of co-variables in blocks controlled by gender and age according to the following sequence: social demography and economic status; family and social context; lifestyle and health conditions (Figure 1). Models were built with samples from each community (*quilombola* and *non-quilombola*), and all the co-variables that presented associations with the results, with significance levels under 20% in the bivariate analysis, were included in the initial models. A level of significance under 5% was used in all the tests and for the permanence of variables in the final model. The models were compared through the Akaike criterion, and the adequacy of the predicted values was assessed by the chi-square test, since the models were nested and had different covariables, and followed a chi-square distribution.

To evaluate the effect of losses on the outcome, the natural expansion factors were calibrated (28). Overweight estimates were compared using the test of proportions for the entire sample and each stratum. The Stata program, version 15.0 (Stata Corporation, College Station, USA), was used for data analysis.

## Results

The study interviewed 390 adolescents; 167 lived in *quilombola* and 223 lived in *non-quilombola* communities, with losses of 15.2 and 7.9%, respectively. The losses varied with sex, with a higher prevalence in males for *non-quilombola* adolescents (*p* = 0.038). However, the estimated outcomes, with and without the calibration factor for this variable for the entire sample and for each stratum, did not present significant differences. Therefore, the variable was considered in the analyses performed.

**Figure 2:**
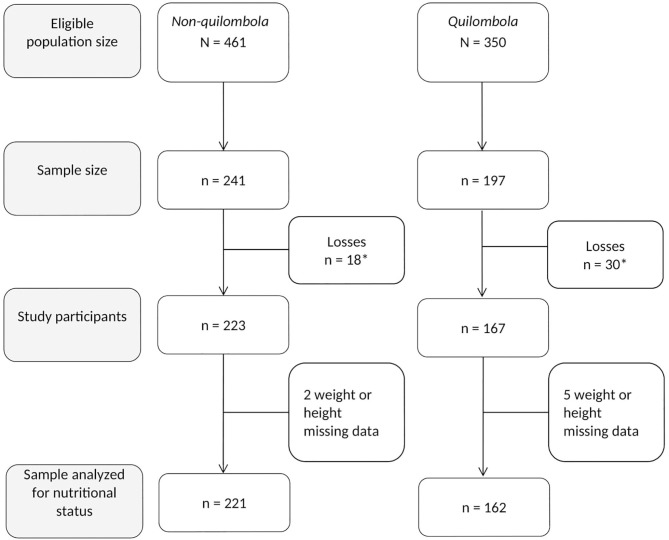
Flow-chart of the final sample for the *quilombola* and *non-quilombola* stratum. Research *Adolescer*, Bahia, 2015. *Losses due to: closed household, adolescent not found after three visits and refusals.

Of the 390 adolescents who participated in the research, data on the nutritional status were missing in seven (three losses for height and four for weight), resulting in a total sample size of 383 adolescents. Among them, 162 (42.3%) were from *quilombola* communities and 221 (57.7%) were from *non-quilombola* communities (Figure 2).

**Figure 3:**
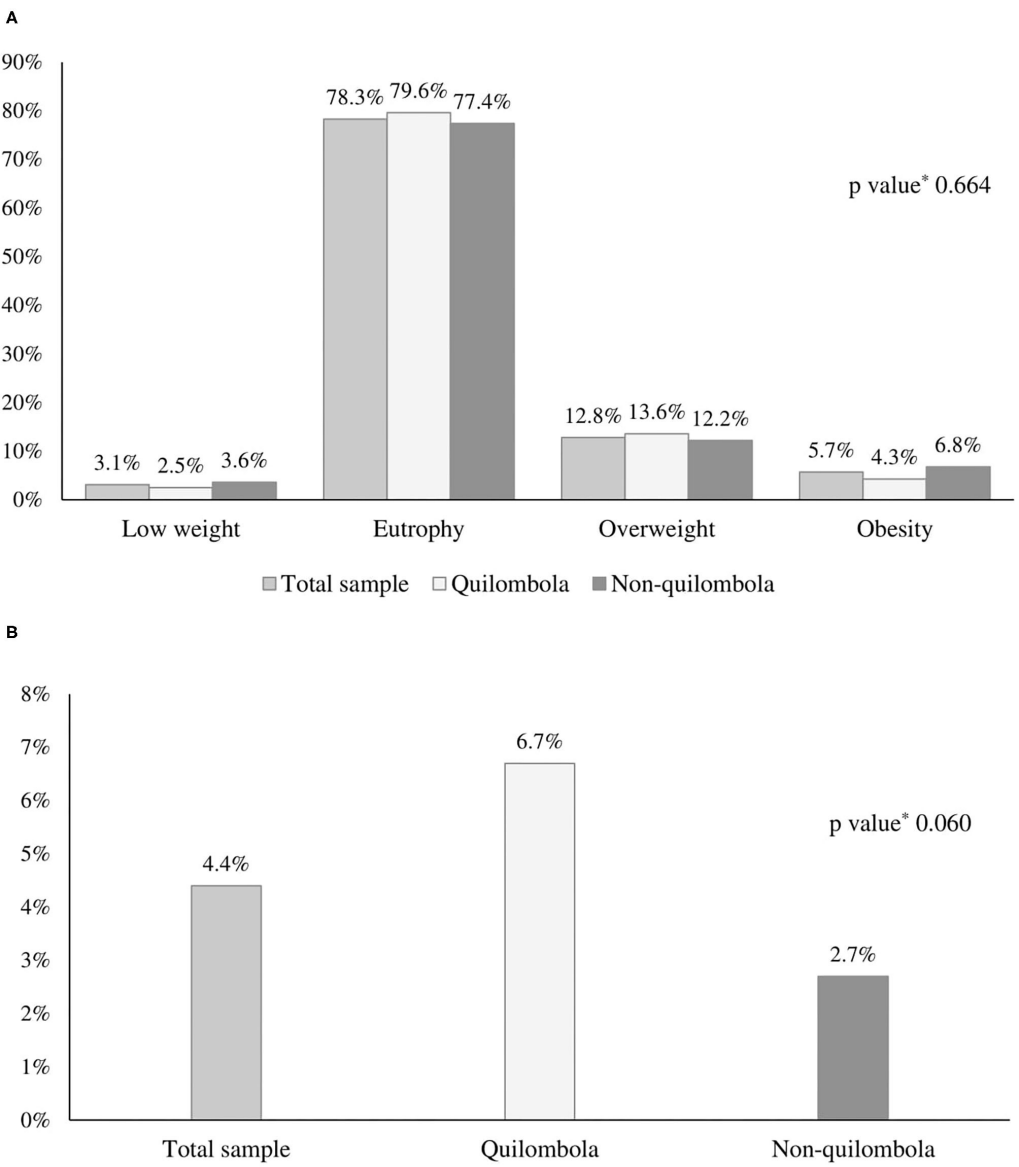
Nutritional status (*A* – *n* = 383) and occurrence of height deficit (*B* – *n* = 387) of adolescents from a rural area of the Northeast of Brazil. Research *Adolescer*, Bahia, 2015. *p value calculated by Pearson's chi-squared test to compare *quilombola* and *non-quilombola*.

Among the participants, 18.5% (95% CI: 14.9–22.8%) presented overweight (overweight/obesity)−17.9% (95% CI: 12.7–24.6%) were *quilombolas* and 19.0% (95% CI: 14.3–24.8%) *non-quilombolas*. Height deficiency occurred in 4.4% (95% CI: 2.7–6.9%) of rural adolescents−6.7% (95% CI: 3.7–11.7%) were *quilombolas* and 2.7% (95% CI: 1.2–5.9%) were *non-quilombolas* (Figure 3).

In the bivariate analysis, the prevalence of overweight was significantly higher among adolescents with stationary screen time (PR: 1.58; 95% CI: 1.03–2.41). The prevalence was lower in those who attended school (PR: 0.50; 95% CI: 0.30–0.83) and had the habit of having breakfast regularly (PR: 0.59; 95% CI: 0.36–0.96). The *quilombola* adolescents who attended school had a lower prevalence of overweight (PR: 0.33; 95% CI: 0.17–0.63). No association was observed between overweight and explained variances among *non-quilombolas* (Table 1).

**Table 1:** Overweight among *quilombola* and *non-quilombola* adolescents, according to studied variables, from a rural area of the Northeast of Brazil (*n* = 383).

**Variances**	**Total sample**				***Non-quilombola***				***Quilombola***			
	***n* (%)**	***p*-value**	**PR**	**95% CI**	***n* (%)**	***p*-value**	**PR**	**95% CI**	***n* (%)**	***p*-value**	**PR**	**95% CI**
**Living in** ***quilombola*** **community**		0.784										
No	42 (19.0)		1.00	–			–				–	
Yes	29 (17.9)		0.94	0.61–1.45								
**Economic level**		0.147				0.160				0.625		
B/C	33 (22.2)		1.00	–	25 (22.7)		1.00	–	8 (20.5)		1.00	–
D/E	38 (16.2)		0.73	0.48–1.12	17 (15.3)		0.67	0.39–1.18	21 (17.1)		0.83	0.40–1.73
**Gender**		0.124				0.125				0.558		
Male	29 (15.4)		1.00	–	17 (15.0)		1.00	–	12 (16.0)		1.00	–
Female	42 (21.5)		1.40	0.91–2.14	25 (23.2)		1.54	0.88–2.69	17 (19.5)		1.22	0.62–2.40
**Age**		0.404				0.100				0.674		
≤12 years old	29 (22.1)		1.00	–	21 (26.6)		1.00	–	8 (15.4)		1.00	–
13–15 years old	18 (15.8)		0.71	0.42–1.21	10 (15.4)		0.58	0.29–1.14	8 (16.3)		1.06	0.43–2.61
≥16 years old	24 (17.4)		0.79	0.48–1.28	11 (14.3)		0.54	0.28–1.04	13 (21.3)		1.39	0.62–3.09
**Race/color**		0.877				0.454				0.417		
Not black	16 (18.0)		1.00	–	10 (15.9)		1.00	–	6 (23.1)		1.00	–
Black	55 (18.7)		1.04	0.63–1.72	32 (20.3)		1.28	0.67–2.44	23 (16.9)		0.73	0.33–1.63
**School years**		0.086				0.142				0.562		
Under 5 years old	29 (19.5)		1.00	–	16 (19.3)		1.00	–	13 (19.7)		1.00	–
6–9 years old	35 (21.1)		1.08	0.70–1.70	21 (22.8)		1.18	0.66–2.12	14 (18.9)		0.96	0.49–1.90
10 years old or more	6 (9.0)		0.46	0.20–1.06	4 (8.90)		0.46	0.16–1.30	2 (9.1)		0.46	0.11–1.90
**Family composition**		0.760				0.568				0.409		
Lives with parents	48 (18.4)		1.00	–	27 (17.3)		1.00	–	21 (20.0)		1.00	–
Lives with father or mother	18 (20.5)		1.11	0.68–1.81	11 (22.5)		1.30	0.69–2.42	7 (18.0)		0.90	0.41–1.95
Does not live with parents	5 (14.7)		0.80	0.34–1.87	4 (25.0)		1.44	0.58–3.62	1 (5.6)		0.28	0.04–1.95
**Currently attending school**		0.012^*^				0.538				0.005^*^		
No	12 (34.3)		1.00	–	4 (23.5)		1.00	–	8 (44.4)		1.00	–
Yes	59 (17.0)		0.50	0.30–0.83	38 (18.6)		0.79	0.32–1.96	21 (14.6)		0.33	0.17–0.63
**Experience with bullying**		0.356				0.396				0.413		
Never	45 (18.2)		1.00	–	25 (17.4)		1.00	–	20 (19.4)		1.00	–
Rarely/sometimes	19 (17.3)		0.95	0.58–1.54	13 (20.6)		1.19	0.65–2.17	6 (12.8)		0.66	0.28–1.53
Often/always	7 (29.2)		1.60	0.81–3.15	4 (30.8)		1.77	0.73–4.32	3 (27.3)		1.40	0.49–4.00
**Number of close friends**		0.761				0.920				1.000		
Until 2	11 (17.2)		1.00	–	7 (18.4)		1.00	–	4 (15.4)		1.00	–
3 or more	60 (18.8)		1.09	0.61–1.96	35 (19.1)		1.04	0.50–2.16	25 (18.4)		1.19	0.45–3.16
**The practice of physical activity**		0.598				0.450				0.929		
Active	31 (17.4)		1.00	–	17 (16.8)		1.00	–	14 (18.2)		1.00	–
Inactive	40 (19.5)		1.12	0.73–1.71	25 (20.8)		1.24	0.71–2.16	15 (17.7)		0.97	0.50–1.88
**Stationary screen time**		0.034†				0.068				0.257		
No	31 (14.8)		1.00	–	18 (14.8)		1.00	–	13 (14.8)		1.00	–
Yes	40 (23.3)		1.58	1.03–2.41	24 (24.5)		1.66	0.96–2.88	16 (21.6)		1.46	0.75–2.85
**Regular intake of unhealthy food**		0.634				0.908				0.540		
No	26 (19.9)		1.00	–	14 (19.4)		1.00	–	12 (20.3)		1.00	–
Yes	45 (17.9)		0.90	0.58–1.39	28 (18.8)		0.97	0.54–1.72	17 (16.5)		0.81	0.42–1.58
**Breakfast intake**		0.040^*^				0.206				0.137		
No	15 (28.9)		1.00	–	8 (27.6)		1.00	–	7 (30.4)		1.00	–
Yes	56 (16.9)		0.59	0.36–0.96	34 (17.7)		0.64	0.33–1.25	22 (15.8)		0.52	0.25–1.08
**The habit of eating while watching TV**		0.509				0.607				0.109		
No	44 (19.6)		1.00	–	24 (17.9)		1.00	–	20 (22.2)		1.00	–
Yes	27 (17.0)		0.86	0.56–1.33	18 (20.7)		1.16	0.67–2.00	9 (12.5)		0.56	0.27–1.16

*Research Adolescer, Bahia, 2015*.

*n (%), absolute and relative frequency; p-value calculated by Pearson's chi-square or Fisher's exact test; PR, prevalence ratio; 95% CI, 95% confidence interval*.

*Economic level: B and C, R$ 1,277.00 (US$ 481.89) to R$ 6,006.00 (US$ 2,266.41) average gross family income/month; D and E, up to R$ 895.00 (US$ 337.73) average gross family income/month. Estimate US commercial dollar values based on the quote currency on December 31, 2014 (US$ 1.00 = R$ 2.65)*.

*Statistically significant (p < 0.05)*.

*Associated with a higher prevalence of overweight*.

* *Associated with a lower prevalence of overweight*.

In the multivariate-adjusted model, overweight among rural adolescents was negatively associated with age ≥16 years (PR: 0.51; 95% CI: 0.28–0.95), the habit of having breakfast regularly (PR: 0.58; 95% CI: 0.35–0.98), and the condition of attending school (PR: 0.35; 95% CI: 0.17–0.71). Stationary screen time increased the occurrence of overweight (PR: 1.61; 95% CI: 1.05–2.46). For *quilombolas*, overweight remained associated with attending school (PR: 0.26; 95% CI: 0.09–0.69). In the adjusted model for *non-quilombolas*, despite the absence of statistical significance, stationary screen time (PR: 1.61; 95% CI: 0.92–2.83) was important to explain the results (Table 2).

**Table 2:** Factors associated with the occurrence of overweight, according to multivariate analysis, for the total sample, *non-quilombola* and *quilombola*.

**Variances**	**Total sample**		***Non-quilombola***		***Quilombola***	
	**PR**	**95% CI**	**PR**	**95% CI**	**PR**	**95% CI**
**Gender**						
Male	1.00	–	1.00	–	1.00	–
Female	1.17	0.76–1.81	1.38	0.78–2.44	1.29	0.67–2.48
**Age**						
≤12 years old	1.00	–	1.00	–	1.00	–
13–15 years old	0.70	0.42–1.19	0.55	0.28–1.07	1.04	0.42–2.58
≥16 years old	0.51	0.28–0.95^*^	0.53	0.28–1.02	0.77	0.27–2.16
**Breakfast intake**						
No	1.00	–	–	–	–	–
Yes	0.58	0.35–0.98^*^	–	–	–	–
**Stationary screen time**						
No	1.00	–	1.00	–	–	–
Yes	1.61	1.05–2.46^†^	1.61	0.92–2.83	–	–
**Currently attending school**						
No	1.00	–	–	–	1.00	–
Yes	0.35	0.17–0.71^*^	–	–	0.26	0.09–0.69^*^

*Research Adolescer, Bahia, 2015*.

*PR, adjusted prevalence ratio; 95% CI, 95% confidence interval*.

*Statistically significant (p < 0.05)*.

† *Associated with a higher prevalence of overweight*.

* *Associated with a lower prevalence of overweight*.

## Discussion

The present study evidenced a low prevalence of overweight among rural adolescents, in line with data recorded previously for the adolescent population in Brazil (23.7%) (8). Aspects such as having breakfast regularly, attending school, and being in the age group ≥16 years reduced the prevalence of overweight, whereas stationary screen time increased the prevalence. Attending school reduced the prevalence, specifically, among *quilombola* adolescents.

A cross-sectional study that used data from *III Pesquisa Estadual de Saude e Nutricao (PESN)* (III State Research of Health and Nutrition) carried out in 2006 showed that 13.3% of children and adolescents of Pernambuco in the Northeast of Brazil were overweight (29). Ramires et al. (30) found a higher prevalence of overweight/obesity (24.0%) among children and adolescents between 5 and 19 years of age in the Northeast. Similar to our study, Cordeiro et al. (31) showed that 17.2% of *quilombola* children and adolescents registered in urban and rural schools in 12 cities in Goias in the Midwest of Brazil were overweight.

International studies showed a great variation in the percentage of overweight adolescents. Kułaga et al. (32) researched on school children and adolescents in Poland and found that 19.4% boys and 13.0% girls were overweight. López-Sánchez et al. (33) evaluated children and adolescents living in Southern Europe between 7 and 19 years of age and estimated that 37.3% were overweight. Results obtained in three cross-sectional studies on children and adolescents in 1985, 1995, and 2005 in China showed that the prevalence of overweight and obesity was significantly higher in urban adolescents aged between 13 and 18 years (2.7, 10.9, 19.1%, respectively) compared to the rural ones (0.6, 2.5, 10.1%, respectively). However, another Chinese cross-sectional study in 2014 displayed a substantial increase in the prevalence of overweight in rural areas (10.1% in 2005 to 17.1% in 2014) in comparison to urban areas (19.1% in 2005 to 19.5% in 2014) (34).

Sedentary lifestyle and the lack of physical activity are the most important risk factors for noncommunicable chronic diseases, such as obesity, cardiovascular diseases, hypertension, and diabetes mellitus (35). Despite the adolescent being classified as active in our study, an association between sedentary behavior and poor lifelong health conditions may prevail (36). Sedentary behavior in relation to stationary screen time is defined as the time a resting individual spends in front of a screen, including TV, computer monitor, cellphone, and tablet (26). In this study, the stationary screen time increased the prevalence of overweight by 61% in rural adolescents.

Due to engrossing technological advances, adolescents often replace active leisurely activities with resting activities related to screen time (37). The PeNSE (2015) showed that around 60% of Brazilian students have the habit of watching TV for more than 2 h on a weekday (8). This leads to overweight and obesity because of a lower calorie burn and higher intake of high-calorie food (38, 39). In Brazil, overweight children and adolescents often indulge in sedentary behavior (40). In rural areas, besides social and economic vulnerabilities, the lack of an appropriate environment for physical activities can contribute to a higher screen time and a consequent weight gain among adolescents (41).

Prevention of unhealthy behavior was important to avoid overweight. The importance of regular physical activity, reducing sedentariness, and a healthy diet should be highlighted. Besides, attending school lowered the prevalence of overweight by promoting a healthy lifestyle and changing inadequate behaviors through education (42). In Brazilian schools, *Base Nacional Comum Curricular (BNCC)*—the National Common Curricular Basis—elaborated by the Ministry of Education, allocates designated spaces and promotes physical education classes to encourage physical activities in children and adolescents (43). WHO advises adolescents to practice moderate- to high-intensity physical activities for 60 min or more per day (25). This can be achieved with an appropriate calorie burn during the physical education classes occurring in opposite shifts, especially during the sports matches and championships promoted by schools. However, a new law states that high school students can be exempted from mandatory physical education classes. This will gradually reduce the rights and contribute negatively to the nutritional status of adolescents.

In contrast, the rural scenario and their physical activities are relevant factors for this study. A study on adolescents in Pernambuco, a Brazilian Northeastern state, presented higher levels of physical activities, lower preference for passive leisure, and lower sedentariness in rural adolescents than in urban ones. Rural adolescents often take up jobs earlier and involve themselves in activities that require physical strength in the countryside/agriculture and household chores; these contribute to their active lifestyles (44). Our research on the rural *quilombola* and *non-quilombola* communities revealed poor access to public transportation and poorly accessible roads; these drive them to resort to other modes of transport including walking or riding a bicycle. These may be associated with the lower prevalence of overweight among rural adolescents.

Regarding healthy nutritional habits, the regular intake of breakfast reduced the prevalence of overweight by 42% in rural adolescents. This practice is also related to a more regular consumption of meals and reducing the habit of snacking on high-carb foods throughout the day (45). Besides, healthy diet and weight control have been associated with a low intake of fat and balanced intake of grains, fruits, and dairy at breakfast (46, 47).

According to Sousa et al. (16), adolescents from the same rural regions investigated in the present study presented healthier food intake and diet than urban adolescents. The *quilombola* adolescents had a high intake of beans but a low intake of more expensive foods, like milk, vegetables, and fruits, which correlate with the higher vulnerability of these communities by impacting their access to a healthier and more varied diet (16).

Concerning the school environment, in the public school, rural adolescents have access to a subsidized diet with good nutritional quality as per the *Programa de Alimentação Escolar (PNAE)—*School Diet Program—from the Brazilian Ministry of Education. In schools with more vulnerable groups, like the indigenous Brazilians and *quilombolas*, a different budget is calculated and food is offered in the elementary school with the aim of improving the nutritional status and valuing the diet culture of these adolescents (48).

*Programa de Saude na Escola (PSE)*—Health at School Program—is also a Brazilian public policy that influences schools and aims at developing projects that help children and adolescents to face vulnerabilities in their daily lives (49). Therefore, a partnership between the health and education sectors is an important way to reduce the main health risk factors because many actions that promote adolescents' health still do not cover their needs (50).

The adolescents' age can also influence their choices, life habits, and developmental changes of the body and metabolism. According to our results, adolescents who are ≥16 years are 49% less overweight compared to the younger ones; this was in accordance with the trends observed in Brazilian adolescents where a higher prevalence of overweight was observed among younger adolescents between 13 and 15 years of age (25.1%) (8). Another study on abdominal obesity showed that the older adolescents (13–15 and 16–19 years of age) had an inverse correlation with abdominal obesity; older adolescents showed a lower prevalence of abdominal overweight (51). Boricic et al. (52) seconded this with evidence suggesting that overweight reduced as age increased in adolescents considered in their study, in both genders.

Growth spurts are characteristic of the adolescence period and work against fat gain. They result in a consequent increase of bone and muscles mass (53). These metabolic alterations can help lower the prevalence of overweight, accompanied with the adoption of a healthy lifestyle. Besides these, older adolescents may have already taken up jobs. Working in jobs that require greater physical activity reduces sedentariness.

The prevalence of overweight (18.5%) and height deficit (4.4%) was low among rural adolescents in our study. However, the characteristics of an incomplete nutrition transition process were not examined. A nutritional deficit may show a progressive and meaningful reduction while there is a gradual increase in overweight/obesity (30, 54). The high speed of nutrition transition was evidenced by Azzopardi et al. (55) when they identified a 120% increase of overweight or obese cases in adolescents worldwide between 1990 and 2016. Abarca-Gómez et al. (7) mentioned that an unhealthy nutrition transition can contribute to height deficit and overweight in children, adolescents, and adults, resulting in a high BMI and poor lifelong health conditions. A study with data from the *Pesquisas Estaduais de Saude e Nutricao no Pernambuco* (1997 and 2006)—State Researches on Health and Nutrition in Pernambuco—showed that even with the reduction of height deficit during this period, there was a significant percentage (10.9%) of adolescents with height issues (56).

Even without assessing malnutrition history in our study, the height deficit in these communities indicate past malnutrition. This is because compromised nutritional status, including the low intake of specific nutrients, is one of the most significant determiners of height deficit (57). Besides that, the height deficit is usually associated with poor social and economic conditions. Therefore, height deficit can be considered a useful sign to demonstrate the health conditions of a population (58). The *quilombola* adolescents who were assessed showed a higher prevalence of height deficit (6.7%) compared to the *non-quilombolas* (2.7%), and although not statistically significant, this difference reemphasizes the worrying scenario of vulnerability among *quilombola* communities who continue to be exposed to racial and ethnical discrimination. Another study conducted in these communities showed that despite the absence of malnutrition, food shortage still exists among *quilombola* families in the Northeast. This may have a negative influence on the health, perspectives, and behaviors of these adolescents (13).

### Study Strengths and Limitations

This study features a comprehensive population as, assesses health aspects of communities that are traditionally vulnerable, mainly from the social and economic points of view and their accessibility to health policies and services. Certain methodological aspects of this study, such as (i) sample calculation that guarantees valid estimates for both groups (*quilombolas* and *non-quilombolas*); (ii) partnership with health teams of the region, allowing access to residences that resulted in a fewer number of refusals, show that our results could be broadly applicable.

Nevertheless, our study has some limitations. As this is a cross-sectional study, it is not possible to infer the temporal nature of some observed associations. We also did not consider the stage of sexual maturation of adolescents [as measured by the Tanner scale (59)], which may have resulted in fewer discrepancies. However, BMI curves of age and gender that we used as nutritional status marks are commonly used in population studies and are recommended by the WHO.

## Conclusion

The adolescents in the study showed a low prevalence of overweight among the *quilombola* and *non-quilombola* rural adolescents. The habit of having breakfast regularly, old adolescents, and attending school negatively influences overweight, while stationary screen time positively influences overweight. Evidence suggests that school is an important space for interventions that improve the quality of life of these individuals, minimizing their vulnerability. In addition, our results reinforce the importance of early adoption of healthy lifestyle habits such as regularly having breakfast and reducing stationary screen time.

Considering the negative consequences of overweight on health, not only in adolescence but also in adulthood, it is still necessary to carry out some actions. These include periodic monitoring of the nutrition status of children and adolescents, providing incentives to encourage the intake of healthy foods and practice of physical activities, and respecting the diversity, culture, beliefs, and dietary habits of the rural populations.

## Data Availability Statement

The raw data supporting the conclusions of this article will be made available by the authors, without undue reservation.

## Ethics Statement

The research was approved by the Institutional Review Board of the Federal University of Bahia (Comite de Etica em Pesquisa com Seres Humanos da Universidade Federal da Bahia—Instituto Multidisciplinar em Saude—Campus Anisio Teixeira), under rule number 639.966. The participants received previous information about the research objectives and data confidentiality, through the reading and signature of Free Informed Consent Form and Informed Consent Form for adolescents under 18 years old. Written informed consent to participate in this study was provided by the participants' legal guardian/next of kin.

## Author Contributions

SC, CT, TdS, and DdM reviewed the literature and had primary responsibility in the final content of this article. SC, CT, TdS, EdS, PM, VB, and DdM wrote the article, analyzed, and interpreted the results. All the authors read and approved the final version of the manuscript.

## Conflict of Interest

The authors declare that the research was conducted in the absence of any commercial or financial relationships that could be construed as a potential conflict of interest.

## References

[1] Salles LMF Infância e adolescência na sociedade contemporânea: alguns apontamentos. Estud Psicol. (2005) 22:33–41. 10.1590/S0103-166X2005000100005

[2] Enes CC Slater B. Obesidade na adolescência e seus principais fatores determinantes. Rev Bras Epidemiol. (2010) 13:163–171. 10.1590/S1415-790X2010000100015 20683564

[3] Azeredo CM de Rezende LFM Canella DS Moreira CR de Castro IR Luiz Odo C Dietary intake of Brazilian adolescents. Public Health Nutri. (2015) 18:1215–24. 10.1017/S1368980014001463 PMC10271650 25089589

[4] Da Costa Louzada ML Baraldi LG Steele EM Martins AP Canella DS Moubarac JC. Consumption of ultra-processed foods and obesity in Brazilian adolescents and adults. Prev Med. (2015) 81:9–15. 10.1016/j.ypmed.2015.07.018 26231112

[5] Goldhaber-Fiebert JD Rubinfeld RE Bhattacharya J Robinson TN Wise PH. The utility of childhood and adolescent obesity assessment in relation to adult health. Med Decis Making. (2013) 33:163–75. 10.1177/0272989X12447240 22647830 PMC3968272

[6] Duncan S Duncan EK Fernandes RA Buonani C Bastos KD-N Segatto AFM. Modifiable risk factors for overweight and obesity in children and adolescents from São Paulo, Brazil. BMC Public Health. (2011) 11:585. 10.1186/1471-2458-11-585 21781313 PMC3154175

[7] Abarca-Gómez L Abdeen ZA Hamid ZA Abu-Rmeileh NM Acosta-Cazares B Acuin C. Worldwide trends in body-mass index, underweight, overweight, and obesity from 1975 to 2016: a pooled analysis of 2416 population-based measurement studies in 128·9 million children, adolescents, and adults. Lancet. (2017) 390:2627–42. 10.1016/S0140-6736(17)32129-3 29029897 PMC5735219

[8] Instituto Brasileiro de Geografia e Estatística Pesquisa nacional de saúde do escolar 2015 (PeNSE 2015). Rio de Janeiro: IBGE (2016). Available online at: https://biblioteca.ibge.gov.br/visualizacao/livros/liv97870.pdf (accessed July 1, 2020).

[9] Food and agriculture organization of the United Nations Rural Poverty in Brasil. FAO (2013). Available online at: http://www.fao.org/3/a-bp560o.pdf (accessed April 9, 2019).

[10] Silva EKP Santos PR Chequer TPR Melo CMA Santana KC Amorim MM. Oral health of quilombola and non-quilombola rural adolescents: a study of hygiene habits and associated factors. Ciênc Saúde Colet. (2018) 23:2963–78. 10.1590/1413-81232018239.02532018 30281734

[11] Oliveira M Ketllin S Caldeira AP Fatores de risco para doenças crônicas não transmissíveis em quilombolas do norte de Minas Gerais. Cad Saúde Colet. (2016) 24:420–7. 10.1590/1414-462x201600040093

[12] Fundação Cultural Palmares Certificação Quilombola. Brasil: Quadro geral por Estados e Regiões (2019). Available online at: http://www.palmares.gov.br/?page_id=37551 (accessed January 22, 2018).

[13] Silva EKP Medeiros DS Martins PC Sousa LA Lima GP Rêgo MAS Insegurança alimentar em comunidades rurais no Nordeste brasileiro: faz diferença ser quilombola? Cad Saúde Públ. (2017) 33:e00005716 10.1590/0102-311x00005716 28591371

[14] Santana KC Teles N Oliveira MHB Medeiros DS Direito à saúde: adolescentes quilombolas em comunidades rurais de Vitória da Conquista (BA). In: Oliveira MHB, Erthal RMC, Vianna MB, Matta JLJ, Vasconcellos LCF, Bonfatti RJ, editors. Direitos Humanos e Saúde: Construindo Caminhos, Viabilizando Rumos, Vol. 1. Rio de Janeiro: CEBES (2017). p. 53–6.

[15] Santos MFS Félix LB Morais ERC Representações Sociais de Juventude em uma Comunidade Quilombola do Agreste Pernambucano. Psico. (2012) 43:12.

[16] Sousa BC Medeiros DS Curvelo MHS Silva EKP Teixeira CSS Bezerra VM Hábitos alimentares de adolescentes quilombolas e não quilombolas da zona rural do semiárido baiano, Brasil. Ciên Saúde Coletiva. (2019) 24:419–430. 10.1590/1413-81232018242.34572016 30726375

[17] Dick B Ferguson BJ Health for the world's adolescents: a second chance in the second decade. J Adolesc Health. (2015) 56:3–6. 10.1016/j.jadohealth.2014.10.260 25530601

[18] Dean AG Sullivan KM Soe MM OpenEpi: Estatísticas Epidemiológicas de Código Aberto para Saúde Pública, Versão. Available online at: www.OpenEpi.com (accessed April 6, 2013).

[19] Instituto Brasileiro de Geografia e Estatística Pesquisa Nacional de Saúde do Escolar 2012 (PeNSE 2012). Rio de Janeiro: IBGE (2013). Available online at: https://biblioteca.ibge.gov.br/visualizacao/livros/liv64436.pdf (accessed August 1, 2018).

[20] Instituto Brasileiro de Geografia e Estatística Pesquisa Nacional de Saúde 2013 (PNS 2013). Questionário do Domicílio. Rio de Janeiro: IBGE (2013). Available online at: https://biblioteca.ibge.gov.br/visualizacao/livros/liv94074.pdf (accessed August 1, 2018).

[21] Ministério da Saúde Orientações para a coleta e análise de dados antropométricos em serviços de saúde: Norma Técnica do Sistema de Vigilância Alimentar e Nutricional (SISVAN). Secretaria de Atenção à Saúde. Departamento de Atenção Básica, Brasília (2011). Available online at: http://bvsms.saude.gov.br/bvs/publicacoes/orientacoes_coleta_analise_dados_antropometricos.pdf (accessed March 18, 2018).

[22] World Health Organization AntropoPlus for Personal Computers Manual: Software for Assessing Growth of the World's Children and Adolescents. Geneva: WHO (2009). Available online at: http://www.who.int/growthref/tools/who_anthroplus_manual.pdf?ua=1 (accessed April 19, 2018).

[23] Onis M Onyango AW Borghi E Siyam A Nishida C Siekmann J. Development of a WHO growth reference for school-aged children and adolescents. Bull World Health Organ. (2007) 85:660–7. 10.2471/BLT.07.043497 18026621 PMC2636412

[24] Associação Brasileira de Empresas de Pesquisa Critério de classificação econômica Brasil 2014 (Base LSE 2012). São Paulo: ABEP (2014). Available online at: http://www.abep.org/criterio-brasil (accessed March 19, 2018).

[25] World Health Organization Global Recommendations on Physical Activity for Health. WHO Guidelines Approved by the Guidelines Review Committee, Geneva: WHO, 2010. Available online at: https://www.who.int/dietphysicalactivity/factsheet_recommendations/en/ (accessed April 9, 2019).

[26] Tremblay MS Aubert S Barnes JD Saunders TJ Carson V Latimer-Cheung AE. Sedentary behavior research network (SBRN)—terminology consensus project process and outcome. Int J Behav Nutr Phys Act. (2017) 14:75. 10.1186/s12966-017-0525-8 28599680 PMC5466781

[27] Levy RB Castro IRR Cardoso LO Tavares LF Sardinha LMV Gomes FS Consumo e comportamento alimentar entre adolescentes brasileiros: Pesquisa Nacional de Saúde do escolar (PeNSE), 2009. Ciên Saúde Coletiva. (2010) 15(Suppl. 2): 3085–97. 10.1590/S1413-81232010000800013 21049149

[28] Szwarcwald CL Damacena GN Complex sampling design in population surveys: planning and effects on statistical data analysis. Rev Bras Epidemiol. (2008) 11(Suppl. 1):38–45. 10.1590/S1415-790X2008000500004

[29] Leal VS Lira PIC Oliveira JS Menezes RCE Sequeira LAS Arruda Neto MS. Overweight in children and adolescents in Pernambuco State, Brazil: prevalence and determinants. Cad Saúde Pública. (2012) 28:1175–82. 10.1590/S0102-311X2012000600016 22666821

[30] Ramires EKNM Menezes RCE Oliveira JS Oliveira MAA Temoteo TL Longo-Silva G. Nutritional status of children and adolescents from a town in the semiarid Northeastern Brazil. Rev Paul Pediatr. (2014) 32:200–7. 10.1590/0103-0582201432309 25479850 PMC4227341

[31] Cordeiro MM Monego ET Martins KA Overweight in Goiás' quilombola students and food insecurity in their families. Rev Nutr. (2014) 27:405–12. 10.1590/1415-52732014000400002

[32] Kułaga Z Grajda A Gurzkowska B Wojtyło MA Gózdz M Litwin MS. The prevalence of overweight and obesity among Polish school-aged children and adolescents. Przegl Epidemiol. (2016) 70:641–51. 28233966

[33] López-Sánchez GF Sgroi M D'Ottavio S Díaz-Suárez A González-Víllora S Veronese N Body Composition in children and adolescents residing in Southern Europe: prevalence of overweight and obesity according to different international references. Front Physiol. (2019) 10:130 10.3389/fphys.2019.00130 30837896 PMC6390201

[34] Zhang YX Wang ZX Zhao JS Chu ZH. Prevalence of overweight and obesity among children and adolescents in Shandong, China: urban–rural disparity. J Trop Pediatr. (2016) 62:293–300. 10.1093/tropej/fmw011 26966244

[35] Pearson N Biddle SJ. Sedentary behavior and dietary intake in children, adolescents, and adults: a systematic review. Am J Prev Med. (2011) 41:178–88. 10.1016/j.amepre.2011.05.002 21767726

[36] Tremblay MS Carson V Chaput J-P Connor Gorber S Dinh T Duggan M. Canadian 24-hour movement guidelines for children and youth: an integration of physical activity, sedentary behaviour, and sleep. Appl Physiol Nutr Metab. (2016) 41(Suppl. 3):S311–27. 10.1139/apnm-2016-0151 27306437

[37] Guerra PH Farias Júnior JC Florindo AA Comportamento sedentário em crianças e adolescentes brasileiros: revisão sistemática. Rev Saúde Públ. (2016) 50:9 10.1590/S1518-8787.2016050006307 PMC4794779 27007685

[38] Camelo LV Rodrigues JFC Giatti L Barreto SM Lazer sedentário e consumo de alimentos entre adolescentes brasileiros: Pesquisa Nacional de Saúde do Escolar (PeNSE), 2009. Cad Saúde Pública. (2012) 28:2155–62. 10.1590/S0102-311X2012001100015 23147957

[39] Oliveira JS Barufaldi LA Abreu GA Leal VS Brunken GS Vasconcelos SML. ERICA: use of screens and consumption of meals and snacks by Brazilian adolescentes. Rev Saúde Públ. (2016) 50(Suppl. 1):7s. 10.1590/S01518-8787.2016050006680 26910539 PMC4767035

[40] Vasconcellos MB Anjos LA Vasconcellos MTL Estado nutricional e tempo de tela de escolares da Rede Pública de Ensino Fundamental de Niterói, Rio de Janeiro, Brasil. Cad Saúde Pública. (2013) 29:713–22. 10.1590/S0102-311X2013000400009 23568301

[41] Müller AW Silva MC Barreiras à prática de atividades físicas de adolescentes escolares da zona rural do sul do Rio Grande do Sul. Rev Bras Ativ Fis Saúde. (2013) 18:344–53. 10.12820/rbafs.v.18n3p344

[42] Araújo C Toral N Silva ACF Velásquez-Melendez G Dias AJR Estado nutricional dos adolescentes e sua relação com variáveis sociodemográficas: Pesquisa Nacional de Saúde do Escolar (PeNSE), 2009. Ciênc Saúde Coletiva. (2010) 15(Suppl. 2):3077–84. 10.1590/S1413-81232010000800012 21049148

[43] Ministério da educação Conselho Nacional de Educação. Base Nacional Comum Curricular (BNCC). Distrito Federal: BNCC (2016). Available online at: http://portal.mec.gov.br/index.php?option=com_docman&view=download&alias=78631-pcp015-17-pdf&category_slug=dezembro-2017-pdf&Itemid=30192 (accessed June 10, 2018).

[44] Regis MF Oliveira LM Santos AR Leonidio ACR Diniz PRB Freitas CMSM. Urban versus rural lifestyle in adolescents: associations between environment, physical activity levels and sedentary behavior. Einstein. (2016) 14:461–7. 10.1590/s1679-45082016ao3788 28076591 PMC5221370

[45] Utley JM Affuso O Rucks AC. Adolescent obesity in contextual settings: a scoping study of multilevel and hierarchical examinations. Clin Obes. (2016) 6:296–304. 10.1111/cob.12163 27627786

[46] Pearson N Biddle SJ Gorely T. Family correlates of breakfast consumption among children and adolescents. A systematic review. Appetite. (2009) 52:1–7. 10.1016/j.appet.2008.08.006 18789364

[47] Riordan F Barret N Michels N Andersen LF Vant Veer P Harrington J Breakfast skipping and overweight/obesity among European adolescents, a cross-selectional analysis of the HELENA dataset: a DEDIPAC study. Revue d'Épidémiologie et de Santé Publique. (2018) 66 (Suppl. 10):S233 10.1016/j.respe.2018.05.007

[48] Ministério da Educação e Cultura Fundo Nacional de Desenvolvimento da Educação. Resolução/CD/FNDE n° 38, de 16 de julho de 2009. Dispõe sobre o atendimento da alimentação escolar aos alunos da educação básica no âmbito do Programa Nacional de Alimentação Escolar (PNAE). Brasília: Diário Oficial da União (2013). Available online at: http://portal.mec.gov.br/index.php?option=com_docman&view=download&alias=8166-res038-16072009-pdf&Itemid=30192 (accessed June 10, 2018).

[49] Ministério da Saúde/Ministério da Educação Caderno do Gestor do Programa Saúde na Escola (PSE). Brasília-DF, (2015). Available online at: http://bvsms.saude.gov.br/bvs/publicacoes/caderno_gestor_pse.pdf (accessed June 10, 2018).

[50] Costa RF Zeitone RCG Queiroz MVO García CIG García MJR. Adolescent support networks in a health care context: the interface between health, family and education. Rev Esc Enferm USP. (2015) 49:741–7. 10.1590/S0080-623420150000500005 26516742

[51] Bozza R Campos W Bacil EDA Barbosa Filho VC Hardt JM. Sociodemographic and behavioral factors associated with body adiposity in adolescents. Rev Paul Pediatr. (2014) 32:241–6. 10.1590/0103-0582201432315 25479856 PMC4227347

[52] Boricic K Simic S Vasiljevic N Marinkovic J Risk factors associated with overweight among adolescents in Serbia/Dejavniki Tveganja, Povezani S Prekomerno Telesno TeŽo Pri Mladostnikih V Srbiji. SJPH. (2014) 53:283–93. 10.2478/sjph-2014-0031 PMC4820196 27669514

[53] Ferriani MGC Santos GVB Adolescência, puberdade e nutrição. Associação Brasileira de Enfermagem. Adolescer. (2001) 1:77–92.

[54] Batista Filho M Assis AM Kac Gilberto Transição Nutricional: conceito e características. In Kac G, Sichieri R, Gigante DP, editors. Epidemiologia Nutricional. 1st ed [Online]. SciELO-Editora FIOCRUZ (2007). p. 580 10.7476/9788575413203

[55] Azzopardi PS Hearps SJC Francis KL Kennedy EC Mokdad AH Kassebaum NJ. Progress in adolescent health and wellbeing: tracking 12 headline indicators for 195 countries and territories, 1990–2016. Lancet. (2019) 393:1101–18. 10.1016/S0140-6736(18)32427-9 30876706 PMC6429986

[56] Leal VS Lira PIC Menezes RCE Oliveira JS Sequeira LAS Andrade SLLS Factors associated with the decline in stunting among children and adolescents in Pernambuco, Northeastern Brazil. São Paulo. Rev Saúde Públ. (2012) 46:234–41. 10.1590/S0034-89102012005000015 22331183

[57] Monteiro CA Benicio MHD Conde WL. Narrowing socioeconomic inequality in child stunting: the Brazilian experience, 1974–2007. Bull World Health Organ. (2010) 88:305–11. 10.2471/BLT.09.069195 20431795 PMC2855601

[58] Pedraza DF Sales MC Menezes TN Fatores associados ao crescimento linear de crianças socialmente vulneráveis do Estado da Paraíba, Brasil. Ciênc Saúde Colet. (2016) 21:935–46. 10.1590/1413-81232015213.20722014 26960105

[59] World Health Organization Physical Status: The Use of and Interpretation of Anthropometry. Report of a WHO Expert Committee. Geneva: WHO, 1995. Available online at: https://apps.who.int/iris/handle/10665/37003 (accessed April 9, 2019).

